# Prevalence of Diarrheagenic *Escherichia coli* (DEC) and *Salmonella* spp. with zoonotic potential in urban rats in Salvador, Brazil

**DOI:** 10.1017/S095026882000285X

**Published:** 2020-11-20

**Authors:** C. Pimentel Sobrinho, J. Lima Godoi, F. Neves Souza, C. Graco Zeppelini, V. Espirito Santo, D. Carvalho Santiago, R. Sady Alves, H. Khalil, T. Carvalho Pereira, M. Hanzen Pinna, M. Begon, S. Machado Cordeiro, J. Neves Reis, F. Costa

**Affiliations:** 1 Biology Institute, Federal University of Bahia, UFBA, Salvador, Brazil; 2 Collective Health Institute, Federal University of Bahia, UFBA, Salvador, Brazil; 3 Department of Wildlife, Fish, and Environmental Studies, Swedish University of Agricultural Sciences, Umeå, Sweden; 4 School of Veterinary Medicine, Federal University of Bahia, UFBA, Salvador, Brazil; 5 Institute of Integrative Biology, University of Liverpool, Liverpool, UK; 6 School of Pharmacy, Federal University of Bahia, UFBA, Salvador, Brazil

**Keywords:** Enterobacteria, *R. norvegicus*, *R. rattus*, odents, Zoonoses

## Abstract

Studies evaluating the occurrence of enteropathogenic bacteria in urban rats (*Rattus* spp.) are scarce worldwide, specifically in the urban environments of tropical countries. This study aims to estimate the prevalence of diarrhoeagenic *Escherichia coli* (DEC) and *Salmonella* spp. with zoonotic potential in urban slum environments. We trapped rats between April and June 2018 in Salvador, Brazil. We collected rectal swabs from *Rattus* spp., and cultured for *E. coli* and *Salmonella* spp., and screened *E. coli* isolates by polymerase chain reaction to identify pathotypes. *E. coli* were found in 70% of *Rattus norvegicus* and were found in four *Rattus rattus*. DEC were isolated in 31.3% of the 67 brown rats (*R. norvegicus*). The pathotypes detected more frequently were shiga toxin *E. coli* in 11.9%, followed by atypical enteropathogenic *E. coli* in 10.4% and enteroinvasive *E. coli* in 4.5%. From the five black rats (*R. rattus*), two presented DEC. *Salmonella enterica* was found in only one (1.4%) of 67 *R. norvegicus*. Our findings indicate that both *R. norvegicus* and *R. rattus* are host of DEC and, at lower prevalence, *S. enterica*, highlighting the importance of rodents as potential sources of pathogenic agents for humans.

Synanthropic rodents of the species *Rattus norvegicus* (urban brown rat) and *Rattus rattus* (black rat) are of great importance in public health for being reservoirs of a diversity of pathogens [1]. They are hosts of *Leptospira interrogans*, *Streptobacillus moniliformis* and Seoul virus (SEOV) [1]. Moreover, they carry ectoparasites such as fleas, which act as reservoirs of *Yersinia pestis*, *Rickettsia typhi* and *Bartonella* spp. [1, 2]. All those microorganisms cause diseases of great public health importance, where humans become infected via direct contact, food or environmental contamination [1].

In temperate regions, studies have identified enterobacteria of clinical importance, such as *Salmonella* spp. and *Escherichia coli*, in these rodent species, which can be eliminated through faeces and be another source of infection for humans [3, 4]. Urban rats are likely to acquire such bacteria from the environment [4]. Identical antibiotic-resistant and virulent *E. coli* strains were found in samples from rodents captured in agricultural facilities, in domestic animals and environmental samples from the same facilities [3]. Moreover, rats living on commercial farms also carry the same *Salmonella* spp. strains that are detected in resident chickens [5].

Diarrheagenic *E. coli* (DEC) and *Salmonella* are important sources of foodborne diseases and gastroenteritis in humans [4]. *Salmonella* was the aetiologic agent most prevalent (92.2%) in 12 503 foodborne disease outbreaks in Brazil, as reported by the Information System for Notifiable Diseases (SINAN) from 2000 to 2017 [6]. Herein, we (a) estimate the prevalence of DEC and *Salmonella* in *R. norvegicus* and *R. rattus* from urban tropical slums in the city of Salvador, Brazil, determine the susceptibility profile of *Salmonella* isolates to antimicrobials, and identify *E. coli* pathotypes isolated from urban rats faeces.

We live-trapped *R. norvegicus* and *R. rattus* in four slum communities within the Suburban Sanitary District of the city of Salvador, Brazil, from April to June 2018. Approximately 30% of the populations of Salvador (and Brazil) reside in similar low-income and poor environmental conditions [7]. The sampled areas ranged from 0.07 to 0.09 km^2^. Within each community, 40 randomised points were selected as trapping points, in which two Tomahawk traps were set with fresh sausage for four nights and checked early morning. Traps with individuals of *R. norvegicus* and *R. rattus* were placed in plastic bags and transported to the Ambulatory of Wild Animals at the Federal University of Bahia, where individual rats were anaesthetised and humanely sacrificed. We recorded body weight, length, sex and reproductive status for each animal. We stratified *R. norvegicus* functional groups according to weight, and juveniles were classified as <200 g, subadults between 200 and 399 g and adults as ≥400 g [2]. In females, sexual maturity was determined by the presence of a scar on the placenta, pregnancy (observation of embryos) and evidence of lactation. In males, maturity was determined by the presence of scrotal testicles. Faeces were collected through the rectal swab. The swabs were transported in Cary Blair [8] medium and forwarded to the Laboratory of Microbiology of Research, Faculty of Pharmacy at Federal University of Bahia.

The samples from the swab were cultured on MacConkey's agar medium (MC), Hektoen enteric agar (HE) and in selenite cystine broth (SC) followed by incubation at 35–36°C for 18–24 h. After incubation, dishes were evaluated by the presence of lactose fermenting colonies (lac+) and lactose non-fermenting colonies (lac−). Isolated colonies were sent to biochemistry identification through assay kit EPM – MILI – CITRATO (LABORCLIN). The bacteria previously identified as *Salmonella* and *E. coli* were submitted to agglutination tests in lamina for serological characterisation (somatic and flagellar antigens) following fabricator's instructions (PROBAC DO BRASIL, São Paulo). The antiserum used was as follows: flagellar anti-antigen (H) and somatic anti-antigen (O) for *Salmonella* spp., anti-*E coli* polyvalent invader A, anti-*E. coli* polyvalent B and anti-O157 enterohaemorrhagic for *E. coli* strains. The isolates of *E. coli* were submitted to resistance screening for ceftriaxone (30 μg) (cephalosporins) and ertapenem (10 μg) (carbapenemics) through disk-diffusion method. The samples identified as *Salmonella* sp. were sent to the automated system Vitek^®^ (bioMérieux) for confirmation of the species and susceptibility profile to antimicrobials.

DNA extraction was performed with an isolate from *E. coli* of all positive rats in the culture. For this, four or five bacterial colonies from those isolates were suspended in ultra-purified water in a sterile microtube. Thereafter, the microtubes were put in a water bath at 100°C for 5 min. The lysates were centrifuged for 2 min at 10 000 rpm. After, 50 μl of the supernatant (DNA) was collected to be used in polymerase chain reaction (PCR). The PCR was performed in order to detect the presence of six genes used to distinguish three *E. coli* pathotypes: *eae*, *bfpA* and *bfpB* to enteropathogenic *E. coli* (EPEC), *eae*, *stx1* and *stx2* for shiga toxin *E. coli* (STEC), *ipaH* for enteroinvasive *E. coli* (EIEC). The reaction's conditions and the primers used are described in Supplementary Table S1. The control strains for each gene were: O55:H7 for *eae*, *bfpA* and *bfpB* (EPEC), C1845 for *stx1*, EDL 933 for *stx2* (both STEC) and 012NM for *ipaH* (EIEC) gene. The reactions were performed using GoTaq Green master mix (Promega, Madison, WI, EUA) 0.34 μM of each primer and 2.0 μl of DNA. The PCR products were analysed by agarose gel electrophoresis 2% with Tris-borate-EDTA buffer (TBE), the lines were detected through ethidium bromide staining (EtBr).

Demographic variables and trapping location for trapped *R. norvegicus* (sex, developmental stage and neighbourhood) were stratified by presence of *E. coli* with virulence gene (*vs. E. coli*- negative for virulence genes). We tested for association between the presence of *E. coli* and these factors using *χ*^2^ test and Fisher's exact test. All hypothesis tests of Pearson's correlation were performed with a significance level of *P* = 0.05. *R. rattus* individuals were not demographically classified or statistically analysed due to the number of captures.

We captured 72 rats, 67 (93.1%) were *Rattus norvegicus* and five (6.9%) were *Rattus rattus*. Among *R. norvegicus*, 40 (59.7%) were females and 27 (40.3%) males. Sexual maturity could be analysed in 58 rats (*R. norvegicus*), of which 40 (69.0%) were sexually active. The functional groups were distributed as follows, six (9.0%) were adults, 37 (55.2%) were subadults and 24 (35.8%) were juvenile. The mean weight was 262.5 g (interquartile range (IQR) = 157.5–335.0 g) and the mean body length (nose–anus) was 213.0 mm (IQR = 186.25–224.25 mm). The presence of cutaneous wounds or scars was observed in 54 (80.6%) animals.

*E. coli* was found in 47/67 (70.1%) of *R. norvegicus* and were found in four out of five in *R. rattus*. Serological characterisation was performed in 51 isolates of *E. coli*, four (two from *R. norvegicus* and two from *R. rattus*) showed positive agglutination for Polyvalent A group (Anti O28ac, O29, O136, O114, O152) and 12 (only in *R. norvegicus*) showed positive agglutination for Polyvalent B group (Anti O112ac, O124, O143, O164, O167). Only one (in *R. norvegicus*) isolate of *E. coli* agglutinated for *E. coli* O157 enterohaemorrhagic. All of the isolates from *E. coli* were sensitive to ceftriaxone (30 μg) and ertapenem (10 μg) by disk-diffusion test. *Salmonella enterica* subsp. *enterica* was found in only one (1.4%) of 67 *R. norvegicus*. This strain was resistant to cefalotin, cefuroxime, cefuroxime axetil, amikacin and gentamicin.

Diarrheagenic *E. coli* was detected in 21/67 (31.3%) of *R. norvegicus*. Regarding sex, developmental stage and capture neighbourhood, the data about *E. coli* pathotypes prevalence in *R. norvegicus* are demonstrated in Table 1. The DEC categories detected more frequently were STEC (*n* = 8; 11.9%), followed by atypical enteropathogenic *E. coli* (aEPEC) (*n* = 7; 10.4%) and EIEC (*n* = 3; 4.5%). Three (4.5%) of the isolated were detected as hybrid strains, since they presented common genes to two pathotypes: two strains were positive for *stx*2 and *ipaH* genes, and one for *stx*1 and *ipaH*. In relation to STEC occurrence, the *stx*2 gene was detected more frequently (*n* = 4; 6.0%), followed by *stx*1 (*n* = 3; 4.5%), *stx*2 and *eae* (*n* = 3; 4.5%), and lastly *stx*1 and *eae* less frequently (*n* = 1; 1.5%).

**Table 1.** Pathotypes prevalence of *E. coli* in *R. norvegicus* by developmental stage, sex and neighbourhood of capture

			No. of positive rats (proportion^a^)				
Categories		No. of rats	*E. coli* positive	aEPEC	STEC	EIEC	STEC + EIEC
General		67	21 (31.3%)	7 (10.4%)	8 (11.9%)	3 (4.5%)	3 (4.5%)
Developmental stage	Juvenile	24	2 (8.3%)	0 (0.0%)	1 (4.2%)	1 (4.2%)	0 (0.0%)
	Subadult	37	15 (40.5%)	7 (18.9%)	6 (16.2%)	1 (2.7%)	1 (2.7%)
	Adult	6	4 (66.7%)	0 (0.0%)	1 (16.7%)	1 (16.7%)	2 (33.3%)
Sex	Male	27	10 (37.0%)	5 (18.5%)	2 (7.4%)	1 (3.7%)	2 (7.4%)
	Female	40	11 (27.5%)	2 (5.0%)	6 (15.0%)	2 (5.0%)	1 (2.5%)
Neighbourhood	Alto do Cabrito	19	8 (42.1%)	3 (15.8%)	5 (26.3%)	0 (0.0%)	0 (0.0%)
	Marechal Rondon	9	3 (33.3%)	1 (11.1%)	0 (0.0%)	2 (22.2%)	0 (0.0%)
	Rio Sena	10	4 (40.0%)	2 (20.0%)	2 (20.0%)	0 (0.0%)	0 (0.0%)
	Nova Constituinte	20	5 (25.0%)	1 (5.0%)	0 (0.0%)	1 (5.0%)	3 (15.0%)
	São Marcos	2	1 (50.0%)	0 (0.0%)	1 (50.0%)	0 (0.0%)	0 (0.0%)
	Federação	7	0 (0.0%)	0 (0.0%)	0 (0.0%)	0 (0.0%)	0 (0.0%)

a Percentages refer to positive animals (of each test) divided by tested animals in each category (row).

There were no differences in the prevalence of DEC in male and female rats (37.0% and 27.5%), respectively, (*χ*^2^ = 0.22, *P* = 0.63). Through evaluation of developmental stage, pathogenic *E. coli* was found in four (66.7%) adults, 15 (40.5%) subadults and two (8.3%) juvenile, being significantly higher in subadults relative to juvenile (*P* = 0.008). We did not identify the differences in DEC prevalence between neighbourhoods (*P* ≥ 0.32) (Table 1). Out of the four black rats (*R. rattus*) found with *E. coli,* two of them presented DEC. One strain was STEC and another EIEC. Other enterobacteria were identified in the present report whose prevalence is in Supplementary Tables S2 and S3.

We detected a high prevalence of DEC in *R. norvegicus* and *R. rattus* from urban tropical slums, as well as the occurrence of *S. enterica* in *R. norvegicus*. The bacteria found have implications for human health, and rats can be a source of those bacteria, especially for the residents of communities where the rats were captured. In our results, DEC was detected in 31% of the brown rats, presenting genes associated with intestinal pathogenicity in humans. Correspondingly, 17% of isolated from *R. norvegicus* in Berlin presented genes associated with extra-intestinal pathogenicity [9]. For developmental stage, our data suggested that age it is, in fact, a risk factor to have DEC. The DEC pathotype more frequently found was STEC (12%), however, no strains were found with the three characteristic genes as the previous study by Vancouver, which 4% of the urban rats were positive for STEC (*stx*1, *stx*2 e *eae*) [4]. The STEC strains that presented only *stx*2 (6.0%) were more frequent in this study. It is noteworthy that isolates of STEC that produce only *stx*2 are more often associated with serious diseases, such as haemolytic−uraemic syndrome (HUS), than those isolates producing only *stx*1 or *stx*1 associated to *stx*2 [10]. *S. enterica* subsp. *enterica* was found in 1% of *R. norvegicus*. This finding is similar to what has been related in other studies [4, 8].

One limitation of this study was that only one isolate from *E. coli* by rat was submitted to screening for resistance to cephalosporin and carbapenem, therefore, this may have underestimated the presence of resistance to cephalosporin and carbapenem in the rats analysed. Moreover, complementary phenotypic tests or molecular techniques for detection of extended-spectrum *β*-lactamase (ESBL) was not investigated. Another limitation is that only one isolate of *E. coli* from each rat was tested for the pathotype presence, thus the presence of more than one pathogenic *E. coli* in the same rat cannot be discarded. Lastly, our low sample size limited the ability to perform stratified analysis among the demographic variables (sex, developmental stage and neighbourhood). Nevertheless, the presented analysis of enteropathogenic bacteria in urban rats is a pioneer in Brazil.

Rats from Salvador can spread strains of DEC and *Salmonella enterica*, which makes these rodents even more important sources of zoonotic agents of public health importance. Moving forward, comprehensive investigations are required to clarify the ecology and epidemiology of these diseases, as well as the impact on the health of residents who have direct and/or indirect contact with these animals.

The Animal Using Ethics Committee (CEUA) (protocol no. 27/2017) of the Federal University of Bahia approved all methods and procedures animal.

## Data Availability

The data that support the findings are available on request.

## Supplementary material

For supplementary material accompanying this paper visit http://dx.doi.org/10.1017/S095026882000285X. click here to view supplementary material
