# Supplementary material for: Prevalence of Diarrheagenic Escherichia coli (DEC) and Salmonella spp. with zoonotic potential in urban rats in Salvador, Brazil
Source: Epidemiol Infect. 2020 Nov 20;149:e128. doi: 10.1017/S095026882000285X (PMC8167902; doi:10.1017/S095026882000285X)
Supplement: Supplementary file 1 [file S095026882000285Xsup001.doc]

**SUPPLEMENTARY MATERIAL**

Table S1 – PCR primers sequences used to identification of the marker genes of *E. coli* pathotypes.

| **Gene** | **Sequence (5´-> 3´)** | **Size (pb)** | **Annealing temperature (ºC)** | **Reference** |
| --- | --- | --- | --- | --- |
| *eae* | CTGAACGGCGATTACGCGAA  CCAGACGATACGATCCAG | 917 | 52 | Reid et al., 1999 |
| *bfpA* | AATGGTGCTTGCGCTTGCTGC GCCGCTTTATCCAACCTGGTA | 326 | 52 | Torniepo RTH et al., 1995 |
| *bfpB* | GACACCTCATTGCTGAAGTCG  CCAGAACACCTCCGTTATGC | 910 | 63 | Müller et al., 2007 |
| *stx1* | ATAAATCGCCATTCGTTGACTAC  AGAACGCCCACTGAGATCATC | 180 | 52 | Paton and Paton, 1998 |
| *stx2* | GGCACTGTCTGA  AACTGCTCC  TCGCCAGTTATCTGACATTCTG | 255 | 49 | Paton and Paton, 1998 |
| *ipaH* | GTTCCTTGACCGCCTTTCCGATAC CGTC  GCCGGTCAGCCACCCTCTGAGAG TAC | 619 | 52 | Toma et al., 2003 |

Table S2 – Enterobacteria prevalence by genus and specie of *R. norvegicus* of Salvador-BA (n=67).

| **Genus** | **Nº of rats (%)** | **Species** | **Nº of rats (%)** |
| --- | --- | --- | --- |
| *Citrobacter* | 26 (38.8 %) | *Citrobacter freundii* | 21 (31.3 %) |
|  |  | *Citrobacter diversus* | 3 (4.5 %) |
|  |  | *Citrobacter amalonaticus* | 1 (1.5 %) |
|  |  | *Citrobacter youngae* | 1 (1.5 %) |
| *Enterobacter* | 10 (14.9 %) | *Enterobacter cloacae* | 7 (10.4 %) |
|  |  | *Enterobacter agglomerans* | 2 (3.0 %) |
|  |  | *Enterobacter gergoviae* | 1 (1.5%) |
| *Klebsiella* | 12 (17.9 %) | *Klebsiella pneumoniae* | 6 (9.0 %) |
|  |  | *Klebsiella cozaenae* | 1 (1.5 %) |
|  |  | *Klebsiella aerogenes* | 5 (7.4 %) |
| *Serratia* | 6 (9.0 %) | *Serratia marcescens* | 3 (4.5 %) |
|  |  | *Serratia liquefaciens* | 3 (4.5 %) |
| *Proteus* | 3 (4.5 %) | *Proteus vulgaris* | 2 (3.0 %) |
|  |  | *Proteus mirabilis* | 1 (1.5 %) |

Table S3 – Enterobacteria prevalence by genus and specie of *R. rattus* of Salvador-BA (n=5).

| **Genus** | **Nº of rats (%)** | **Species** | **Nº of rats (%)** |
| --- | --- | --- | --- |
| *Citrobacter* | 2 (40.0 %) | *Citrobacter freundii* | 2 (40.0 %) |
| *Enterobacter* | 3 (60.0 %) | *Enterobacter cloacae* | 2 (40.0 %) |
|  |  | *Enterobacter gergoviae* | 1 (20.0 %) |
| *Kluyvera* | 1 (20.0 %) | *Kluyvera cryocrescens* | 1 (20.0 %) |
